# Application of Spray Drying Particle Engineering to a High-Functionality/Low-Solubility Milk Thistle Extract: Powders Production and Characterization

**DOI:** 10.3390/molecules23071716

**Published:** 2018-07-14

**Authors:** Francesca Sansone, Tiziana Esposito, Maria Rosaria Lauro, Patrizia Picerno, Teresa Mencherini, Franco Gasparri, Stefania De Santis, Marcello Chieppa, Claudia Cirillo, Rita Patrizia Aquino

**Affiliations:** 1 Department of Pharmacy, University of Salerno, via Giovanni Paolo II, 132, 84084 Fisciano, Italy; tesposito@unisa.it (T.E.); ppicerno@unisa.it (P.P.); tmencherini@unisa.it (T.M.); info@gasparrifranco.it (F.G.); aquinorp@unisa.it (R.P.A.); 2 PhD Program in Drug Discovery and Development, University of Salerno, via Giovanni Paolo II, 132, 84084 Fisciano, Italy; mchieppa@unisa.it; 3 IRCCS “de Bellis”, Laboratory of Experimental Immunopathology, 70013 Castellana Grotte, Italy; s-desantis@live.it; 4 EBRIS, European Biomedical Research Institute of Salerno, 84121 Salerno, Italy; 5 Department of Industrial Engineering and NANO_MATES Research Centre, University of Salerno, via Giovanni Paolo II, 132, 84084 Fisciano, Italy; clcirillo@unisa.it

**Keywords:** silymarin complex, water solubility, in vitro dissolution and permeation tests, anti-inflammatory activity, functional stability

## Abstract

Many natural compounds having antioxidant and anti-inflammatory activity are a potential target for new therapies against chronic inflammatory syndromes. The oral administration of functional herbal supplements may become a prevention strategy or therapy adjuvant for susceptible patients. A case study is our milk thistle (*Silybum marianum*) extract rich in silymarin complex. A water-soluble microencapsulated powder system was developed by a spray drying technique to improve the poor silymarin bioactivity after oral administration. Sodium carboxymethylcellulose (NaCMC) was employed as coating/swelling polymer matrix and sodium lauryl sulfate (SLS) as the surfactant (1:1:0.05 *w*/*w*/*w*). A H_2_O/EtOH/acetone (50/15/35 *v*/*v*/*v*) solvent system was used as liquid feed. The microsystems were capable of improving the in vitro dissolution and permeation rates, suggesting an enhancement of bioactivity after oral administration. The microsystems protect the antioxidant activity of silymarin after harsh storage conditions period and do not affect the anti-inflammatory properties of the raw extract (efficient already at lower concentrations of 0.312 mg/mL) to reduce dendritic cells (DCs) inflammatory cytokine secretion after lipopolysaccharide administration. This approach allows managing particle size, surface properties and release of bioactive agents improving the bioactivity of a herbal supplement and is also possibly applicable to many other similar natural products.

## 1. Introduction

Plant extracts intake has been associated with numerous health benefits, but their efficacy is often limited due to insufficient dose efficiency after oral administration. To be consumed as part of a daily diet or as a dietary supplement, the absorption and bioavailability of nutraceuticals must sustain a significant therapeutic level when orally ingested [1]. The compound must be dissolved or dispersed in the aqueous intestinal lumen, to become accessible for absorption. A way to enhance the absorption after the oral administration as well as, targeted biological efficacy is to produce technological delivery systems able to improve dissolution and permeation rate of the active ingredient [2,3]. Numerous delivery systems had been developed and studied extensively in oral drug delivery [4,5,6], but the application of this research area to nutraceuticals only is emerging very recently [7,8,9,10,11,12]. The milk thistle (*Silybum marianum* (L.) Gaertn, Asteraceae), extract is an essential source of silymarin; it is a well-known complex of flavonolignans (silybin A and B, isosilybin A and B, silydianin, and silychristin) and flavonoids (taxifolin and quercetin) used to treat a variety of ailments like liver (hepatitis, cirrhosis, and icterus), kidney, and gallbladder problems [13,14]. Silybin is the primary and most active component of silymarin complex; it is a potent antioxidant able to reduce inflammation, also having membrane-stabilizing, hepatoprotectant, anticarcinogenic and antiviral activities [15]. Silymarin also has got significant anti-cancerous properties towards prostate cancer, but it is inadequately utilized for cancer therapy due to its hydrophobic nature and poor bioavailability [16]. The extensive phase II metabolism, low permeability across epithelial cells, low aqueous solubility, and rapid excretion in bile and urine [15]. These are characteristics that make it unstable and poorly absorbed from gastrointestinal tract. These factors need a form of silymarin able to overcome absorption problems [13,17]. During past years several approaches have been used to augment Silymarin bioavailability after oral administration such as complexation with phospholipids [18], niosomes [19] and liposomes [20,21,22], inclusion complex with β-cyclodextrins [23], incorporation in solid dispersions [24], formation of derivatives (salt, prodrug, and glycosides) as well as micronization and nanonisation [25]. In a recent work, silymarin poly(d,l-lactic-co-glycolic acid) (PLGA) nanoparticles (NPs) have been developed to improve the therapeutic efficacy of silymarin towards prostate cancer by single emulsion solvent evaporation technique [16]. Conventional liposomes, mainly composed of phospholipids and cholesterol, are susceptible to disintegration due to the effects of the gastric acid, pancreatic lipase, and intestinal bile salts; thus, more recently, stable hybrid liposomes-encapsulated silymarin [26] and novel vesicular nanosystem bilosomes loading silymarin have been produced and compared to conventional liposomes, aiming at increasing the hepatoprotective activity of the drug [27]. 

The selection of technique and compositional materials to produce the delivery system plays an essential role in developing a functional, safe and marketable product [28,29]. On this trend, microencapsulation by spray drying technique is a cost-effective one-step process as compared to other encapsulation methods [23]. It is a micro-/ nanoencapsulation technique mainly used in food and pharmaceutical trade in processing materials very rapidly while providing relative control of the final product properties [30,31]. In this study, we produced a water-soluble spray-dried powder to encapsulate the silymarin rich milk thistle extract. Technological characteristics (process yield, loading efficiency, solid state) as well as in vitro dissolution and permeation behavior of microencapsulated extract have been investigated to unprocessed one. The silymarin content and antioxidant activity of the new herbal supplement were evaluated before and after a storage period under harsh conditions.

The anti-inflammatory activity of the extract after the process transformation was performed in vitro cultured dendritic cells (DCs) exposed to microencapsulated or raw extract. Intestinal DCs are the immune cells most frequently exposed to nutritional derived products. Differently, from DCs resident in non-mucosal tissues, intestinal DCs are conditioned by the host and environmental factors to become inflammatory impaired [32]. As DCs precursors become inflammatory impaired once exposed to the intestinal milieu and intestinal DCs can extend process into the intestinal lumen [33,34], we evaluated silymarin abilities to prevent LPS induced inflammatory cytokines secretion, as previously demonstrated for quercetin [35,36,37]. Furthermore, we compared the microencapsulated with to the raw extract showing that microencapsulation does not reduce silymarin anti-inflammatory abilities.

## 2. Results and Discussion

### 2.1. Silymarin Content

The UV Actual Silymarin Content (ASC, expressed as silybin equivalents), of the commercial milk thistle extract (MTE) resulted of 91.06% ± 2.3, while the 29.0% ± 0.8 of the total active content by HPLC was quantified in silybin, in agreement with the producer specifications (silymarin ≥ 90%; silybin A and B > 25%). These high levels of functional active substances have encouraged our research towards the technological improvement of the product to enhance bioavailability after oral intake and to promote its oral administration in in vivo trial. 

### 2.2. Microencapsulation Process 

The obtained results concerning yield, encapsulation efficiency, and particle size have been reported in Table 1. Preliminary experiments on liquid feed preparation have highlighted two issues of particular importance. The MTE raw extract has poor wettability which makes particularly tricky the development of feed suspensions for the spray-drying process (prolonged times of agitation, heating and ultrasound sonication). The non-homogeneous suspension could negatively affect the atomization process (nozzle clogging, pressure drop, flow disruptions) resulting in meager production yield and inadequate particle morphology (coating surfaces routes, partial amorphization, presence of non-encapsulated crystals of extract). A small percentage of SLS as a surfactant (0.005% *w*/*v* of SLS) was directly added to the water medium used to prepare the solvent system to overcome the low wettability of MTE. Safety studies on SLS reported the oral toxic dose (human) at 0.5–5 g/kg [38]. It has been deemed safe by many investigatory pharmaceutical associations in small doses [39]. The concentration of SLS used in our study was calculated by assuming that generally in a unit dose weight of 500 mg for solid dosage form prepared as immediate-release (IR) formulation there are 10 mg of SLS [40]. By the SLS percentage used to prepare the liquid feed (0.005% *w*/*v*), the final formulation may contain a maximum (without considering the process loss) of 2.5 mg SLS in 500-mg unit dose which is very far from the toxic dose. 

The so made suspension could be processed by spray-drying resulting in satisfactory production yield (69.7%). NaCMC was useful as an encapsulant agent leading to an ASC_MTE_ (38.6%) extremely close to the theoretical value (45.5%) which resulted in a high encapsulation efficiency (84.9%). The functionality of MTE is correlated to the silymarin content, the higher the ASC_MTE_ in the produced encapsulated powder form is, the higher should be the final formulation functional activity, thus, the obtained encapsulation efficiency is an exciting and promising characteristic. 

### 2.3. Powder Characterization

#### 2.3.1. Dimensions and Morphology

**Figure 1:**
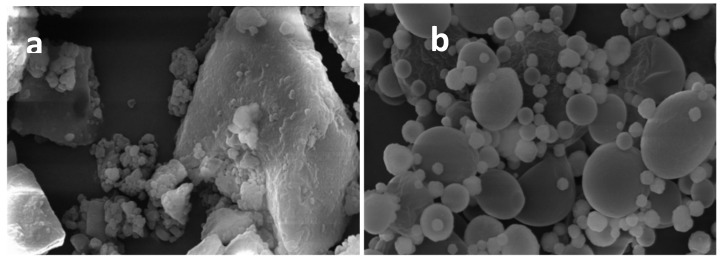
Scanning electron microscopy of MTE raw extract (**a**) and spray dried MTE_mp (**b**).

Results of dimensional analysis (Table 1) indicated that MTE_mp powder system had very narrow size distribution (4.4 µm) to MTE raw extract (19.1 µm). The span value used to describe the distribution width correlating the distribution of particle population, and derived by a model-independent algorithm resulted lower than 3, defining that the volume of particles distribution (distribution width) was in a narrow range. The morphological study showed that MTE raw extract is material in crystalline state (Figure 1a) with high size, irregular shape, and surface; otherwise, MTE_mp (Figure 1b) is formed by small, well-formed, and spherically shaped microparticles with few aggregates and severe reduction in crystallinity. No pores onto the surface, able to promote the loss of the core material, were detected. Decreasing in both particle size and crystallinity increases the surface area exposed to the dissolving solvent and enhance the dissolution rate [41].

**Figure 2:**
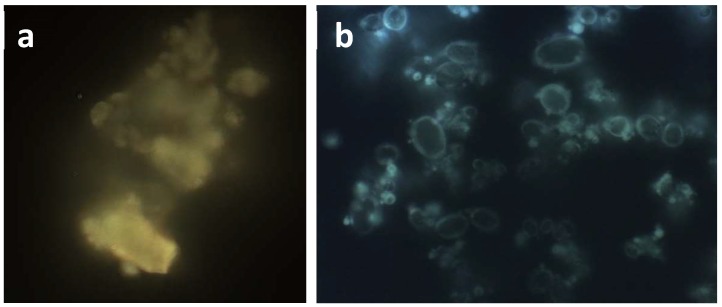
Fluorescence microscopy of crystalline MTE raw extract (**a**) and MTE_mp microparticles (**b**).

FM images (Figure 2) confirmed the crystalline state of MTE raw extract also showing it well embedded within the NaCMC matrix in the MTE mp system. Crystalline raw extract (Figure 2a) has a characteristic yellow fluorescence. MTE_mp microparticles (Figure 2b) show a light blue fluorescence due to flavonoids well embedded within the matrix.

#### 2.3.2. Thermal Analysis, TGA and PXRD

**Figure 3:**
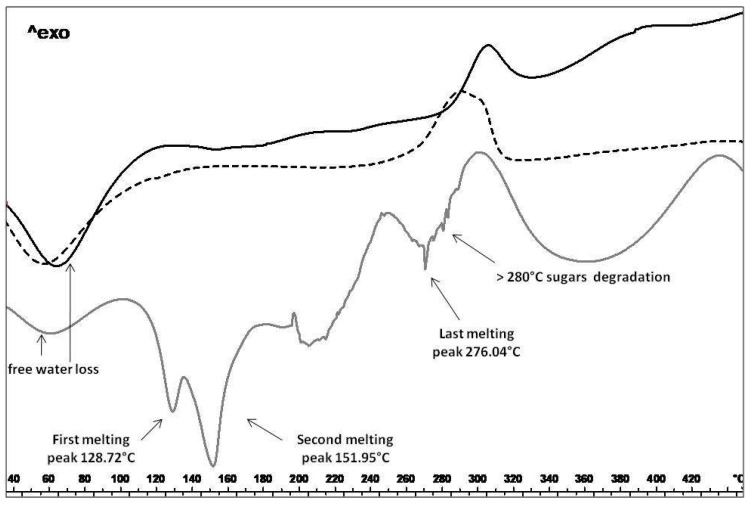
Differential scanning calorimetry analysis. MTE raw extract thermal profile (grey line); MTE_mp (black line) and blank_mp (dotted line).

The variation in the solid state (amorphous/crystalline ratio) of MTE_mp and blank_mp (loaded and unloaded microparticles) to MTE raw extract, was confirmed by DSC (Figure 3). This analysis was also performed to verify the absence of degradation events, possibly occurring during the spray drying process, which could alter the extract functionality. In Figure 3 the thermal trend of MTE raw extract shows the presence of free water (residual humidity; endothermic event around 70 °C), attributable to dehydration of the sample. The two subsequent endothermic events are due to the melting of solid state. In particular, the first endothermic event exhibits a melting point at T = 121.43 °C and a peak visible at T = 128.72 °C. The second endothermic event exhibits a melting point at T = 138.98 °C with a peak visible at T = 151.95 °C. After the last endothermic event at T = 276.04 °C, the final degradation starts at temperatures above 280 °C due to the presence of sugars in the extract. In the thermal profile of MTE_mp powder, the thermal trend of the original material was not detectable, suggesting that MTE was well encapsulated/embedded in the matrix also confirming the good physical interaction with the polymer used as the encapsulant. The spray-drying process, combined with the use of NaCMC and surfactant-water/organic solvent system, allows the conversion of the extract into an amorphous state with a functional interaction in forming homogeneous matrix system. Moreover, no degradation peaks, attributable to chemical or physical instability were detected. 

**Figure 4:**
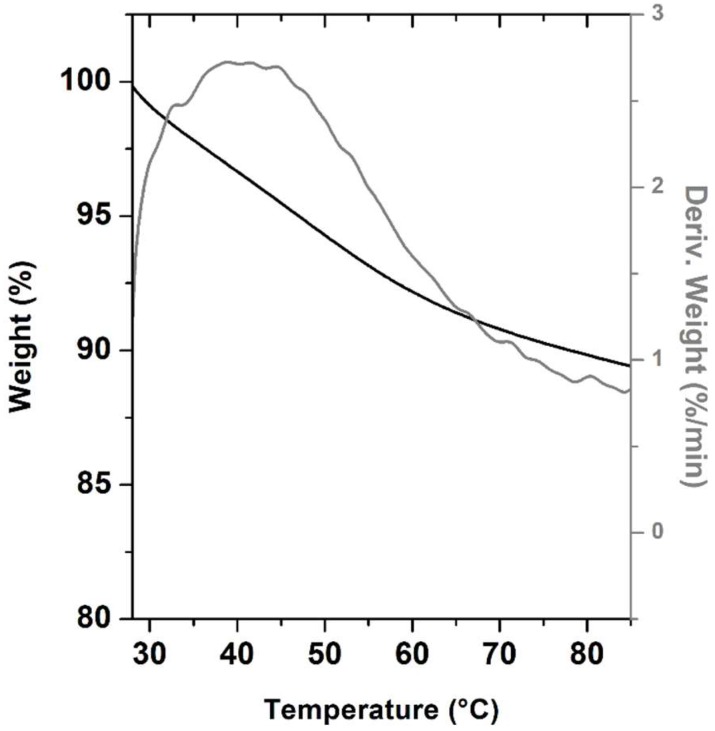
Thermogravimetric analysis. TG-DTG (black line and gray line, respectively) profile of MTE_mp.

To further confirm the dehydration of MTE_mp in the range from room temperature to 85 °C, TGA analysis was also performed. The TG-DTG profile of MTE_mp, reported in Figure 4, exhibits a single weight loss of ~10%. This weigh loss is probably due to residual humidity (loss of free water) still present in the sample.

**Figure 5:**
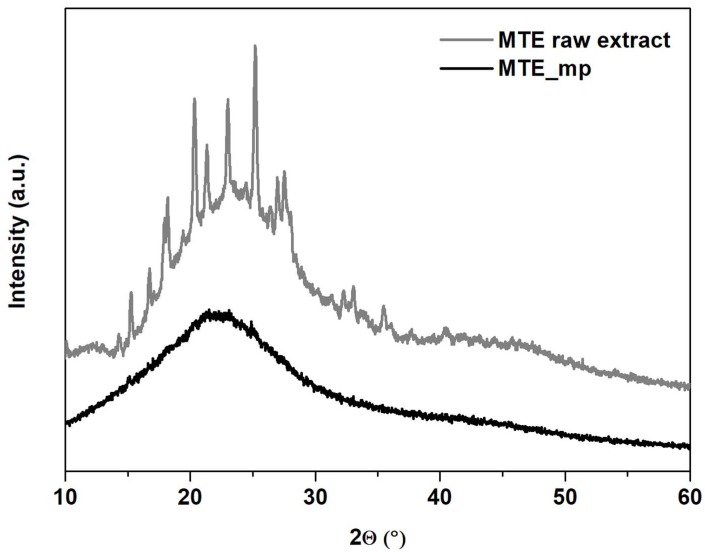
XRD analysis. Diffraction patterns of MTE raw extract (gray line) and MTE_mp (black line).

In agreement with results of SEM and DSC analysisis, MTE_mp powder resulted completely amorphous as indicated by PXRD profiles (Figure 5). The diffraction pattern of the MTE raw extract (Figure 5, gray line) shows, in the range between 12° and about 50° of 2ϑ, a series of typical reflections of crystalline silymarin complex [42]. In the MTE_mp pattern, all the peaks of MTE raw extract disappeared. It exhibits only a unique large hump at about 20° of 2ϑ angle (Figure 5, black line), as typical of amorphous materials. This result indicates that MTE raw extract was successfully encapsulated into MTE_mp microparticles. 

### 2.4. Solubility and Dissolution Profiles

**Figure 6:**
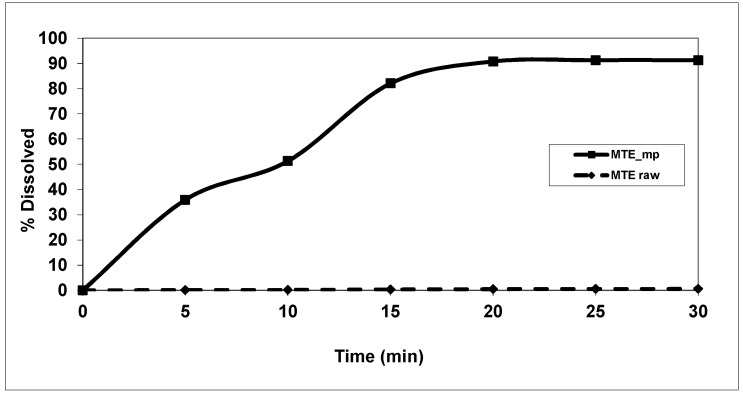
In vitro dissolution profiles of MTE_mp (black line) and MTE extract raw (dotted line) (standard deviation data <1%).

Results from solubility assay (25.5 ± 3.8 mg/L) classified MTE raw extract as a very slightly soluble material [43], and a very slow water dissolution rate was indicated by in vitro dissolution tests (Figure 6). 

After 30 min, the MTE raw extract dissolution rate in water is drastically low, with an average value, graphically expressed as the percentage of dissolution to the time, lower than 1% (about 0.4 mg of raw extract). The dissolution/release profiles of MTE_mp showed a fast enough active content release from the polymeric system with a high increase in dissolution rate until 80% at 15 min and 90% at 30 min to about 0.5% and 1%, respectively, for MTE raw extract. The increase of the microparticle-water interaction, due to both small and amorphous physical state of the material and hydrophilic and water-sorption properties of NaCMC combined with surfactant activity of SLS. 

### 2.5. Permeation Studies

**Figure 7:**
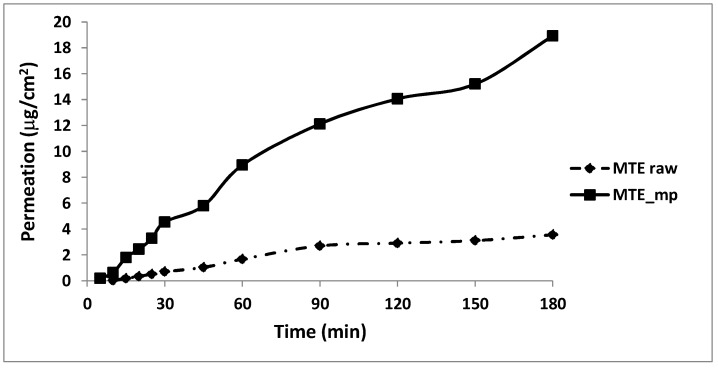
In vitro permeation profile. MTE raw extract (dotted line) and MTE_mp (black line) (standard deviation data <1%).

Transport through membranes is crucial for an active ingredient such as MTE_mp which must cross any bio-membrane surface to be absorbed in vivo after oral or topical administration (Figure 7). Moreover, for in vitro testing of permeation through the membranes, the release of active substances from the ingredient or formulation is often examined, allowing the comparison of formulations or quality control [29,44]. Franz type vertical diffusion with the artificial membrane was used to study the rate of the silymarin release from both MTE raw extract and MTE_mp powder with the aim to provide useful information about the quality of the ingredients and also to estimate how the microencapsulation process and differences in solid state may influence the permeation process through a membrane. The unprocessed extract showed slower silybin permeation (3.6 μg/cm^2^ at 180 min) through membrane than MTE_mp which results in 5-fold higher (18.9 μg/cm^2^ at 180 min) (Figure 7). The amorphous microencapsulated form enhances powder wettability and, therefore, the affinity between the solid and the liquid medium.

### 2.6. Biological Assays

**Figure 8:**
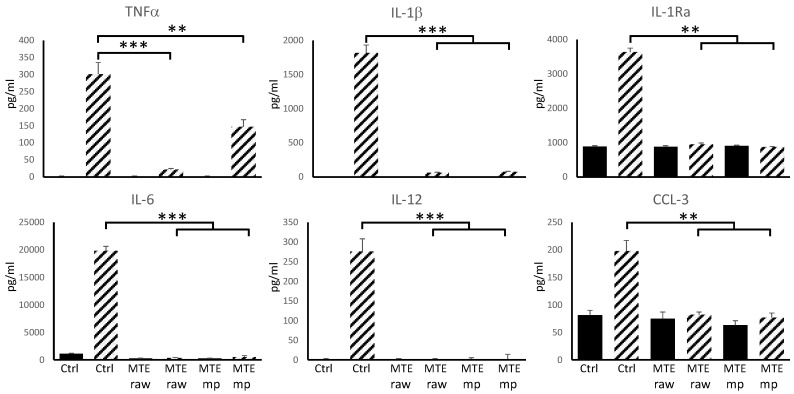
Cytokine secretion by DCs exposed to MTE. Patterned bars represent cytokine concentration in the supernatant of DCs 24 h after LPS administration. DCs exposed to MTE raw material (0.312 mg/mL) or MTE_mp (0.625 mg/mL) fail to produce inflammatory cytokines. MTE exposed DCs in the absence of LPS (filled bars) did not secrete inflammatory cytokines. Data are shown as mean ± S.D. of five independent experiments; ** *p* < 0.01, *** *p* < 0.001.

**Figure 9:**
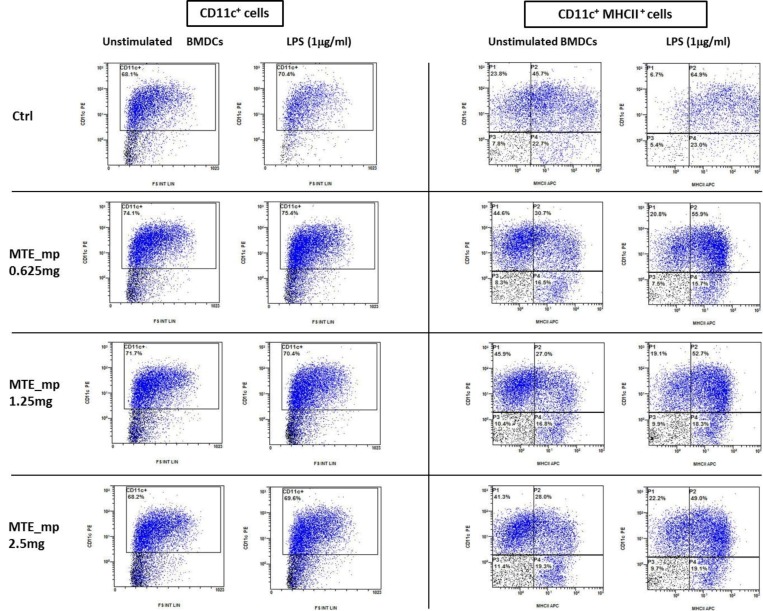
Dendritic cells (CD11C+) do not decrease much even at the highest concentration. MHCII expression decreases after LPS, this is consistent with less maturation and anti-inflammatory effect.

DC progenitors are dynamically regulated to reduce their inflammatory phenotype by the intestinal milieu. DCs tolerance is imprinted by tissutal factors, particularly in an anatomical compartment exposed to a large variety of antigens [45]. In the intestine, nutritional-derived factors contribute to DCs polarization. The anti-inflammatory effects of silymarin have already been demonstrated [46]. Naturally derived compounds, like polyphenols and flavonoids, are a potential target for new therapies against chronic inflammatory syndromes due to their ability to reduce pro-inflammatory cytokine secretion and antigen presentation. We compared the anti-inflammatory properties of raw extract and microencapsulated MTE using in vitro cultured murine dendritic cells exposed to LPS as inflammatory stimulus (Figure 8). DCs were exposed to different MTE raw extract and MTE_mp powder concentrations at day 7, lipopolysaccharide was administered in the culture dish at day 8. 24 h later the supernatant was collected and cells were counted, stained using anti-CD11C and anti-MHCII antibodies and analyzed by FACS (Figure 9). ELISA detected the concentration of inflammatory cytokines released in the supernatant.

Figure 8 shows TNFα, IL-1β, IL-6, and IL-12 reduction following both MTE raw extract and MTE_mp powder administration. 

The secretion of the inflammatory cytokines TNFα, IL-1β, and IL-12 is severely reduced using 0.312 mg/mL of MTE raw extract, while for the MTE_mp it is necessary to reach 0.625 mg/mL. As demonstrated by similar experiments, IL-6 is reduced even by a lower concentration of flavonoids. Finally, the baseline production of the chemokine CCL-3 and the anti-inflammatory IL1-Ra does not appear to be affected by MTE raw extract and MTE_mp exposure, but both compounds prevent the increased secretion observed following LPS administration. DCs exposed to MTE lose their ability to secrete inflammatory cytokine even following LPS administration. 

Here we demonstrate the possibility to reduce TNFα, IL-1β, IL-6, and IL-12 using 0.625 mg/mL of microencapsulated MTE. As reported for other naturally-derived bioactive compounds [36,37] DCs exposure to silymarin microparticles or raw extract prevents LPS-mediated inflammatory cytokine secretion. Apparently, MTE raw extract was already efficient at lower concentration (0.312 mg/mL) than MTE_mp (0.625 mg/mL). This result is depending on the extract content of the MTE_mp formulation (see Table 1). The extract content of MTE_mp is 42.4%, so in 0.625 mg there is about 0.265 mg of extract. This MTE active amount is lower than the effective dosage of MTE raw extract (0.312 mg/mL) meaning an enhancement of the functionality of the extract in microparticulate powder form. Thus, the functionality was positively affected by the process. MTE raw extract is a not dispersible material in water; instead, the innovative amorphous microparticulate MTE_mp avoid this limitation, enhancing the powder wettability also well-affecting the anti-inflammatory properties of the MTE raw extract. This feature makes MTE a water-soluble and drinkable extract, so giving the opportunity to develop new strategies for MTE delivery in clinical treatments.

### 2.7. Stability Studies and Antioxidant Activity

MTE is a rich source of flavonolignans belonging to classe of polyphenols. Unfortunately, these compounds, well known for their broad spectrum of biological properties [14,47,48] react readily with various components and undergo to oxidation/degradation process with consequent functionality decrease. The recommendations of the manufacturer to store silybin standard are to keep it at 4 °C away from light to avoid degradation. Thus, to verify stabilization of encapsulated extract in our microparticle system, accelerated stability studies of MTE_mp powder to MTE raw extract have been performed [49]. After six months, no significant decrease (lower than 1%) in Silybin content was recorded by HPLC method [50]. Moreover, the free-radical scavenging activity, before and after the spray-drying process, and until six months of storage, was evaluated. In the DPPH test, a validated assay for screening the antiradical activity of extracts or natural compounds [17,51], MTE showed a significant dependent-concentration free radical scavenging activity (expressed as EC_50_). The effect remained unaltered after spray drying process (Table 2). Notably, no loss of activity has been observed for MTE-mp powder during six months of harsh storage conditions (Table 2). On the contrary, during the same time, the EC_50_ value of MTE raw extract is going to reduce from 25.2 ± 1.2 μg/mL (0 months) to 71.2 ± 2.9 μg/mL (6 months). This result highlights that NaCMC is a carrier able not only to enhance the solubility properties but also to protect the functionality of the extract. The developed microencapsulation process is efficient to produce long-lasting, stable microparticulate powder systems.

The test was performed until six months of storage. At the time point, three withdrawals for three different samples of each batch were carried out. α-Tocopherol was used as positive control of the DPPH assay. Data are mean ± SD. ** *p* < 0.002 (vs. EC_50_ at t_0_).

## 3. Materials and Methods

### 3.1. Chemicals

Sodium carboxymethylcellulose (NaCMC, medium viscosity, E466) was supplied by Sigma Aldrich (Milan, Italy); Milk thistle extract (MTE) (extract CARDUI MARIANE E FRUCT. SICCUM) code: 345064 batches 248091) standardized at ≥90% in silymarin content (expressed as silybin, calculated on anhydrous substance by HPLC), and Rottapharm/Madaus s.p.a supplied silybin as standard. (Monza, Italy). Sodium lauryl sulfate (SLS) from A.C.E.F. s.p.a. (Piacenza, Italy). All other chemicals used were of reagent grade.

### 3.2. Liquid Feed Preparation and Spray Drying Conditions

A H_2_O/EtOH/acetone liquid system 50/15/35 (200 mL) containing NaCMC and MTE in a 1:1 weight ratio (total amount 2 g), was prepared as follows: NaCMC was dissolved in surfactant-water with 0.005% (g/100 mL) of SLS; then, the organic phase was slowly poured and, finally, the MTE dried extract was added to the polymeric solution under continuous magnetic stirring. A 1% (g/100 mL) total final concentration was reached. The homogeneous suspension was sonicated for 20 min and the liquid feeds were spray dried (Büchi B-191 Mini Spray Dryer; Büchi Laboratoriums-Technik, Flawil, Switzerland) under the following experimental conditions: inlet/outlet temperatures 100/65 °C; spray flow feed rate 5 mL/min; nozzle diameter 0.5 mm; drying air flow 600 L/h, air pressure 6 bar, aspirator 100%. The suspensions were gently stirred using a magnetic stirring to keep homogeneity, during the pumping [29]. As a control, a blank batch only with NaCMC plus SLS dissolved in the solvent system without extract (Blank_mp powder), was prepared. Each preparation was carried out in triplicate. 

### 3.3. Powders Characterization

#### 3.3.1. Quantitative Analysis 

*UV method.* The concentration of silymarin expressed as silybin equivalent, the marker selected for the MTE, was evaluated by measuring absorbance (UV/Vis spectrometer Lambda 25, Perkin Elmer Instruments,Walthan, MA, USA) at λ 288 nm. Calibration curves were previously worked out using MeOH in 1 mm cell (Spectracomp 602, Advanced Products Srl, Milan, Italy) and distilled water in 1 cm cell (Spectracomp 602). Proportionality between absorbance and concentration was verified in the range 50–250 mg/L (*R*^2^ > 0.999) for MeOH and 5–30 mg/L (*R*^2^ > 0.999) for water. 40 mg of all samples were dissolved in 40 mL methanol, shaken and centrifuged for 15 min at 3000× *g*. The supernatants filtered with 0.45 μm filters were analyzed. Each analysis was made in triplicate. 

*HPLC method.* An HPLC apparatus was used to evaluate silybin content (Agilent 1100 series system (Agilent, Santa Clara, CA, USA), equipped with a Model G-1312 pump, a 20 μL Rheodyne Model G-1322A loop (Agilent, Santa Clara, CA, USA), a DAD G-1315 detector, and a 150 × 3.9 mm i.d. C-18 μ-Bondapack column). Peaks areas were calculated with an Agilent integrator. The solvents were HCOOH 0.1% in H_2_O (solvent A) and HCOOH 0.1% in MeOH (solvent B). Elution gradient: 0 → 5 min, 43 → 45% B; 5 → 10 min, 45% B; 10 → 115 min, 45 → 50% B. Analysis was carried out in triplicate: flow rate of 0.8 mL/min; DAD detector set at 288 nm. *Linearity.* Silybin reference standard solutions were prepared at three concentration levels (100–400 µg/mL) and injected three times. The standard curve was analyzed using the linear least-squares regression equation derived from the peak area (regression equation *y* = 2915.3*x* − 63.725, *r^2^* = 0.9998 where *y* is the peak area and *x* the concentration). 

*Specificity.* Peak associated with the marker was identified by its retention time (t_r_ = 26.12 min) and confirmed by co-injection with the standard compound.

#### 3.3.2. Yield and Loading Efficiency

Production yields were gravimetrically determined (balance Crystal 100 CAL – Gibertini Novate, Milanese, Italy, max 110 g d = 0.1 mg; +15 °C/30 °C) and expressed as the weight percentage of the final product compared to the total amount of the materials sprayed.

The theoretical extract content (TEC) was calculated as the percentage of MTE compared to the initial total content of components (MTE plus NaCMC) in the liquid feed before spray-drying.

Actual silymarin content of the unprocessed extract MTE (ASC_MTE_), and of spray-dried microparticles (ASC_MTE_mp_) was determined by UV and HPLC methods as previously described and expressed as silybin equivalents in percentage to 100 mg of powder. 

The actual extract content (AEC) was derived by ASC and calculated as:

AEC% = ASC_MTE_mp_/ASC_MTE_ × 100 (1)

The extract encapsulation efficiency (EE%) was the ratio of the actual to the theoretical extract content:

EE% = AEC/TEC × 100(2)

Each analysis was made in triplicate and results expressed as average values.

#### 3.3.3. Particle Size Analyses 

The dimensional distribution was carried out with a Laser Light Scattering (LLS) granulometer (Beckman Coulter LS 230, Particle Volume Module Plus, Brea, CA, USA). The MTE raw extract was suspended in distilled water; otherwise, microparticles were suspended in isopropanol. About 200 μL of the suspension was poured into the small volume cell to obtain an obscuration between 8 and 12%. Particle size distributions were calculated using the Fraunhofer model. The results were expressed as d_10_, d_50_, and d_90_, indicating the volume diameters at the 10th, 50th and 90th percentiles, of the particle size distribution. The span is defined as:

Span value = (d_90_ − d_10_)/d_50_ (3)

#### 3.3.4. Morphology

Morphology of the particles was examined by scanning electron microscopy (SEM) using an EVO MA 10 microscope (Carl Zeiss, Oberkochen, Germany) with a secondary electron detector (Carl Zeiss SMT Ltd., Cambridge, UK) equipped with a EMSCD005 metallizator (LEICA, Oberkochen, Germany) producing deposition of a 200–400 Å thick gold layer. To verify the microparticles uniformity, at least 20 SEM images were taken into account for each observation.

The fluorescent microscopy assays (FM) were performed observing the samples with a Zeiss Axiophot fluorescence microscope, with 40, 63 and 100 × 1.4 NA Plan Apochromat oil immersion objectives (Carl Zeiss Vision, München-Hallbergmoos, Germany) using standard DAPI (4′,6-diamidino-2-phenylindole) optics that adsorb violet radiation (max 372 nm) and emit a blue fluorescence (max 456 nm).

#### 3.3.5. Differential Scanning Calorimetry (DSC)

Powder samples were analyzed via differential scanning calorimetry on an indium-calibrated Mettler Toledo DSC 822e (Mettler Toledo, Columbus, OH, USA). The instrument automatically determined the blank curve. The baseline correction was previously performed to the sample assays. It was considered as the baseline the separation between the region of latent heat from that of sensible heat; graphically it was the startup deflection proportional to the heat capacity of the sample followed by a DSC curve section with no thermal effects. The integral baseline which takes into account the change in the heat capacity with conversion has been considered. Thermograms were recorded by placing weighed quantities (8–10 mg with a microbalance from MTS Mettler Toledo) of each sample in a 40 μL aluminum pan that was sealed and pierced. The samples underwent one dynamic thermal cycle; they were heated from 25 °C to 450 °C at a heating rate of 10 °C/min.

#### 3.3.6. X-ray Analysis

The solid state of the samples (MTE raw extract and MTE_mp) was analyzed with a D8 X-ray diffractometer using CuKα radiation (*λ* = 1.54050 Å) (Bruker, Milano, Italy) operating at 35 kV and 40  mA. The scanning angle ranged from 10° to 60° of 2ϑ and steps were of 0.0296 of 2ϑ. The diffraction patterns were obtained placing into a sample holder an amount of about 300 mg samples.

#### 3.3.7. Thermogravimetric Analysis

Thermogravimetric analysis (TG-DTG) at 10 °C/min heating rate in air atmosphere from room temperature to 85 °C was performed with a SDT Q600 Analyzer (TA Instruments, New Castle, DE, USA) coupled with a mass spectrometer. The thermograms were obtained using about 10 mg samples in a standard alumina pan. All the experiments were performed in triplicate.

### 3.4. Solubility, Dissolution Tests, and Permeation Profile

All experiments were made in triplicate; only the mean values are reported (standard deviations <1%).

*Solubility.* According to the “Farmacopea Ufficiale della Repubblica Italiana” (FUI), an excessive amount of powder was introduced into glass vials containing 8 mL of H_2_O; the samples were stirred and stored at 25 °C for three days. Then, samples were centrifuged for 15 min at 3.000 rpm, to remove the saturation powder. Supernatants were filtered (0.45 μm filters) and the concentration of the solution was determined by UV method as described before.

*In vitrodissolution/release test.* All the tests carried out in “sink conditions” (<35% of solubility value): samples of 15 mg were dissolved in 1000 mL of distilled water on a dissolution test apparatus n.2: paddle, 75 rpm at 37 °C. Instruments: SOTAX AT Smart Apparatus (Basel, Switzerland) online with a spectrophotometer (UV/Vis spectrometer Lambda 25, Perkin Elmer Instruments, Walthan MA, USA). MTE dissolved or released from MTE microparticles (MTE_mp) was measured by UV method as previously reported. 

*Permeation Profile.* The permeation assays were performed using Franz-type vertical diffusion cells (Hanson Research Corporation, Chatsworth, CA, USA) at 37 °C under continuous stirring (170 rpm) by Teflon-coated lively bars placed in the receptor compartment. Permeation experiments were conducted with Franz cells in a standard configuration using an aqueous suspension (1 µg/µL) containing MTE raw extract or MTE_mp powder applied to the diffusion cell as donor phase. The receptor compartment was filled with 7 mL of distilled water (pH 5.8) and a nitrocellulose membrane (size pores: 0.45 μm) previously set with distilled water was applied between the two compartments (permeation 197 area 1.77 cm^2^). At fixed time, aliquots of 100 μL were analyzed by HPLC for silibinin determination as reported above. The amount of the extract permeated *per* area (Q) for each time interval was calculated using the following Equation (4):

(4) $$Q\left(\frac{mg}{cm^{2}}\right)=\frac{V_{R}\times C_{n}+\sum_{i=0}^{n-1}V_{P}\times C_{i}}{A}$$

where:

*V_R_* is the receiver volume;
*C_n_* is silybin concentration in the receiver at the time n;
*V_P_* is the volume of the removed sample;
*C_i_* is silybin concentration in the receiver at the time n-1;

Permeation data were reported as the quantity of permeated silybin *per* permeation area related to time. 

### 3.5. Generation and Culture of DC

DCs precursors were harvested from murine bone marrow (BM). Briefly, BM from the tibiae and femurs of 6- to 8-week-old male C57BL/6 mice were flushed with RPMI and depleted of red blood cells with ACK cell lysing buffer (GIBCO, Life Technologies, Walthan, MA, USA). Cells were plated in 6-well culture plates (1 × 10^6^ cells/mL; 3 mL/well) in RPMI supplemented with 10% heat-inactivated FBS, 100 U/mL penicillin, 100 mg/mL streptomycin, 25 μg/mL rmGM-CSF, and 25μg/mL rmIL-4 at 37 °C in a humidified 5% CO_2_ atmosphere. On day 3, BMDCs were harvested and plated at 1 × 10^6^/mL in 24-well culture plates. DCs were cultured from murine bone marrow as previously described [37]. On day 7 BMDCs were treated with MTE raw extract and MTE_mp. Lipopolysaccharide (LPS) was administered (1 μg/mL) at day 8 and 24 h later the supernatant (SN) was harvested. 

*Cytofluorimetric analysis.* Cells were stained with Anti-MHC Class II antibodies (clone: M5/114.15.2) and Anti-CD11c-FITC (clone: N418) (Miltenyi Biotec, Bergisch Gladbach, Germany) following manufactures protocol. Flow Cytometer acquisition was performed using NAVIOS (Beckman Coulter, Brea, CA, USA).

### 3.6. Enzyme-Linked Immunosorbent Assay (ELISA)

Cell culture SNs were analyzed to detect the cytokine content. Triplicate of the SN was used to identify TNFα, IL-1β, IL-1Ra, IL-6, IL-12, and CCL-3 concentration using the ELISA kit (R&D Systems, Minneapolis, MN, USA: DY410, DY401, DY480, DY406, DY419, and DY450, respectively), as described by the manufacturer. 

### 3.7. Antioxidant Activity Preservation—Stability Studies

Harsh storage conditions were assessed as reported by the ICH guidelines [50] at 40 ± 2 °C; 75 ± 5% of RH in a climatic chamber (Climatic and Thermostatic Chamber, Mod.CCP37, AMT Srl, Milan, Italy) for six months. At given times (0, 1, 3 and six months) samples of each batch were collected. The free-radical scavenging activity of MTE raw extract and MTE_mp was evaluated over six months of storage using a modified, published method [49]. The first analysis (t_0_) has been conducted after 48 h from the formulation.

*Samples preparation*. After the storage period, 10 mg of MTE raw extract was dissolved in 1 mL of MeOH. Range concentrations of 50–150 μg/mL were tested. 40 mg of MTE_mp were dissolved in 2 mL of MeOH to recover the extract from microparticles. The sample was sonicated 20 min and centrifuged at 4000× *g* for 10 min. The supernatant was dried under vacuum with a rotary evaporator (Rotavapor R-200, Buchi Italia Srl, Cornaredo, Italy). The dried material was dissolved in MeOH and tested at the same concentrations of MTE raw extract.

*Method.* 37.5 μL of the supernatant was added to 1.5 mL of daily prepared 1,1-diphenyl-2-picrylhydrazyl radical (DPPH) solution (0.025 g/L in MeOH). An equal volume (37.5 μL) of the vehicle alone was added to control tubes. Absorbance at 517 nm was measured 30 min after starting the reaction on a Thermo Evolution 201 UV–visible spectrophotometer Thermo Fisher, Walthan, MA, USA). The DPPH concentration in the reaction medium was calculated from a calibration curve analyzed by linear regression, and EC_50_ (mean effective scavenging concentration) was calculated using the Litchfield and Wilcoxon test [44,52] (μg/mL of sample necessary to decrease the initial DPPH concentration by 50%).

## 4. Conclusions

Constant administration of natural anti-inflammatory factors diluted in the drinking water may become a prevention strategy for susceptible patients, also benefit the healthcare system that may reduce the administration of expensive and often not effective treatments. Clinical translation of these results encountered a significant limitation represented by the poor solubility and bioavailability of these compounds. A case study is our milk thistle extract rich in silymarin complex. Extract loaded microparticles by spray drying were produced with high and reproducible yields and encapsulation efficiency. NaCMC used as coating/swelling polymer in a proper solvent system was able to protect the functionality of the extract. The in vitro dissolution and permeation rates of silymarin were dramatically improved suggesting a higher bioavailability after the oral administration. Silymarin anti-inflammatory abilities were preserved by the encapsulation process as demonstrated by the exposure of the immune cells to MTE_mp. Except for a limited increase in the TNFα secretion, silymarin microencapsulated was as efficient as the raw extract concerning inflammatory cytokine suppression, suggesting that the microencapsulation process didn’t affect silymarin efficiency. Our formulation could be the answer to the demand for a functional ingredient which retains its activity over time, easy administrable in the drinking water and bioavailable after the intake. This administration route represents an enormous advantage, especially in the context of nutraceuticals, in intestinal inflammatory syndromes. These results represent the basis for future trials that will validate the efficiency of microencapsulated milk thistle extract administration to prevent or treat chronic inflammatory syndromes.

## Figures and Tables

**Table 1:** Composition and characteristics of raw materials and microparticles.

Samples	Yield %	^a^ TEC %	^b^ TSC %	^c^ ASC %	^d^ AEC %	^e^ EE %	d_50_ μm (span)
NaCMC	-	-	-	-	-	-	21.1 (1.1 ± 0.2)
MTE	-	-	-	91.1 ± 2.3 *	-	-	19.1 (2.5 ± 0.9)
Blank_mp	74.9 ± 3.7 *	-	-	-	-	-	3.9 (1.9 ± 0.4)
MTE_mp	69.7 ± 4.1 *	50	45.5	38.6 ± 0.6 *	42.4 ± 0.6 *	84.9 ± 0.6 *	4.4 (2.7 ± 0.8)

^a^ Theoretical Extract Content; ^b^ Theoretical Silymarin Content; ^c^ Actual Silymarin Content; ^d^ Actual Extract Content; ^e^ Encapsulation efficiency; * Average of triplicate analyses ± standard deviation; Particle size distributions were calculated using the Fraunhofer model and were expressed as d_50_ indicating the volume diameter at the 50th percentile of the particle size distribution and span value derived as [d_90_ − d_10_]/d_50_).

**Table 2:** Free-radical scavenging activity (DPPH test) expressed as the concentration of μg/mL of sample necessary to decrease the initial DPPH concentration by 50% (EC_50_) of the extract before (MTE raw extract) and after (MTE_mp) the microencapsulation process.

Materials	Months			
	0	1	3	6
			*DPPH test EC_50_ µg/mL*	
MTE raw extract	25.2 ± 1.2	51.1 ± 1.3	70.2 ± 1.9	71.2 ± 2.9
MTE_mp	26.3± 0.9	25.1 ± 1.4	26.8 ± 1.0	27.2 ± 1.5
α-tocopherol	10.1 ± 1.3	10.2 ± 1.1	10.1 ± 1.1	10.3 ± 1.2
